# Recent Research Progress in Taxol Biosynthetic Pathway and Acylation Reactions Mediated by *Taxus* Acyltransferases

**DOI:** 10.3390/molecules26102855

**Published:** 2021-05-12

**Authors:** Tao Wang, Lingyu Li, Weibing Zhuang, Fengjiao Zhang, Xiaochun Shu, Ning Wang, Zhong Wang

**Affiliations:** 1 Jiangsu Key Laboratory for the Research and Utilization of Plant Resources, Institute of Botany, Jiangsu Province and Chinese Academy of Sciences (Nanjing Botanical Garden Mem. Sun Yat-Sen), Nanjing 210014, China; johnwt@cnbg.net (T.W.); li13505153082@sina.com (L.L.); weibingzhuang@cnbg.net (W.Z.); fengjiao@cnbg.net (F.Z.); sxc@cnbg.net (X.S.); wangning813@njau.edu.cn (N.W.); 2 Co-Innovation Center for Sustainable Forestry in Southern China, College of Biology and the Environment, Nanjing Forestry University, Nanjing 210037, China

**Keywords:** taxol, biosynthetic pathway, acylation reaction, acyltransferase, latest research progress

## Abstract

Taxol is one of the most effective anticancer drugs in the world that is widely used in the treatments of breast, lung and ovarian cancer. The elucidation of the taxol biosynthetic pathway is the key to solve the problem of taxol supply. So far, the taxol biosynthetic pathway has been reported to require an estimated 20 steps of enzymatic reactions, and sixteen enzymes involved in the taxol pathway have been well characterized, including a novel taxane-10β-hydroxylase (T10βOH) and a newly putative β-phenylalanyl-CoA ligase (PCL). Moreover, the source and formation of the taxane core and the details of the downstream synthetic pathway have been basically depicted, while the modification of the core taxane skeleton has not been fully reported, mainly concerning the developments from diol intermediates to 2-debenzoyltaxane. The acylation reaction mediated by specialized *Taxus* BAHD family acyltransferases (ACTs) is recognized as one of the most important steps in the modification of core taxane skeleton that contribute to the increase of taxol yield. Recently, the influence of acylation on the functional and structural diversity of taxanes has also been continuously revealed. This review summarizes the latest research advances of the taxol biosynthetic pathway and systematically discusses the acylation reactions supported by *Taxus* ACTs. The underlying mechanism could improve the understanding of taxol biosynthesis, and provide a theoretical basis for the mass production of taxol.

## 1. Introduction

Taxol, a kind of tetracyclic diterpenoid secondary metabolite originally extracted from the bark of *Taxus brevifolia* [1], is recognized as one of the most effective antitumor drugs around the world. Taxol could inhibit the division of tumor cells in the G2/M phase of the cell cycle by promoting the formation and stability of microtubules, where it was approved by the U.S. Food and Drug Administration (FDA) for the treatment of several cancers, such as breast, lung and ovarian cancer [2]. Recently, the annual sales of taxol and its related products have exceeded $1 billion [3]. However, the content of taxol in the *Taxus* species is extremely low (the highest content in the bark is only about 0.06%), and the slow growth rate, lack of resources and the poor competitiveness and regeneration ability in the *Taxus* population, resulting in the insufficient yield of natural harvest to meet the growing market demand [4].

Several alternative methods have been explored to increase taxol production. The chemical total synthesis of taxol was reported by Holton et al. [5] and Nicolaou et al. [6] simultaneously with different synthetic routes, but neither of them was commercially viable due to the complex routes, uncontrollable reaction conditions and the low yields [3,7]. The semisynthetic method involves extracting relatively high levels of taxol precursor baccatin III or 10-deacetyl baccatin III (10-DAB) from the reproducible twigs and leaves of cultivated *Taxus* species, and then converting to taxol by chemical synthesis. This is so far the main approach for industrial production of taxol, but it relies heavily on *Taxus* resources in essence [8,9]. Moreover, the extractions of taxol from *Taxus* cell culture and taxol-producing endophytic fungi are also expected, because they are controllable and environmentally sustainable, but no major breakthrough has been achieved at this stage [10,11,12].

Recently, a series of encouraging progress has been made in the production of taxol through heterologous synthesis. Ajikumar et al. [13] reported a multivariate modular pathway engineering, which increased the yield of taxadiene, the first committed taxol intermediate, to approximately 1 g/L in an engineered *Escherichia coli* strain; the yield of taxadien-5α-ol was also up to 58 ± 3 mg/L. Stable coculture of *Saccharomyces cerevisiae* and *E. coli* in the same bioreactor was reported by Zhou et al. [14], who designed specialized environments or compartments for optimal function, and produced 33 mg/L oxygenated taxanes, including a monoacetylated dioxygenated taxane, the taxadien-5a-acetate-10β-ol. Moreover, several plant hosts have also been exploited to produce taxol intermediates, such as *Arabidopsis thaliana* [15], tomato [16] and *Physcomitrella patens* [17]. However, the accumulation of taxadiene could cause the growth retardation and decreased levels of photosynthetic pigment in transgenic plants. Fortunately, Li et al. [18] used a chloroplastic compartmentalized metabolic engineering strategy, which produced taxadiene and taxadiene-5α-ol at 56.6 μg/g FW and 1.3 μg/g FW, respectively, in a high biomass plant *Nicotiana benthamiana*. This study highlights the potential of tobacco as an alternative platform for taxol production. Production of taxol and its precursors in heterologous hosts is more sustainable than extraction from tissues of *Taxus* trees or chemical synthesis. However, it still cannot circumvent the bottlenecks in the taxol biosynthetic pathway. Further developments from diol intermediates to functional taxanes are rarely reported.

The acylation is one of the most important steps involved in modifying the core taxane skeleton, which is mainly catalyzed by a large group of *Taxus*-specific acyl-coenzyme A (acylCoA)-dependent acyltransferases (ACTs) [19]. Interestingly, some of the acylation reactions supported by *Taxus* ACTs were considered the rate-limiting steps in the taxol pathway [20,21]. These corresponding *Taxus* ACT genes have also been important targets for the purpose of overexpression in relevant producing organisms to increase taxol yields [22]. Since the taxol is a highly acylated compound, the less strict acyl or acetyl transfers also contribute to large numbers of taxoid side chain variants possessing distinct biological roles, and theoretically lead to considerable diversion of taxol pathway flux. Therefore, until the entire pathway is characterized, the rate for each step determined, and the regulatory networks understood, any manipulations would be hampered by the lack of knowledge [23].

There is no doubt that the synthesis and yield of taxol will continue to depend on the improvement of biological methods for the foreseeable future. In view of this, the review summarizes the recent advances of taxol biosynthetic pathway, and discusses the acylation reactions mediated by *Taxus* ACTs. The new concepts and perspectives are helpful to develop reliable solutions and provide theoretical basis for producing taxol in large quantities.

## 2. Taxol Biosynthetic Pathway

**Figure 1:**
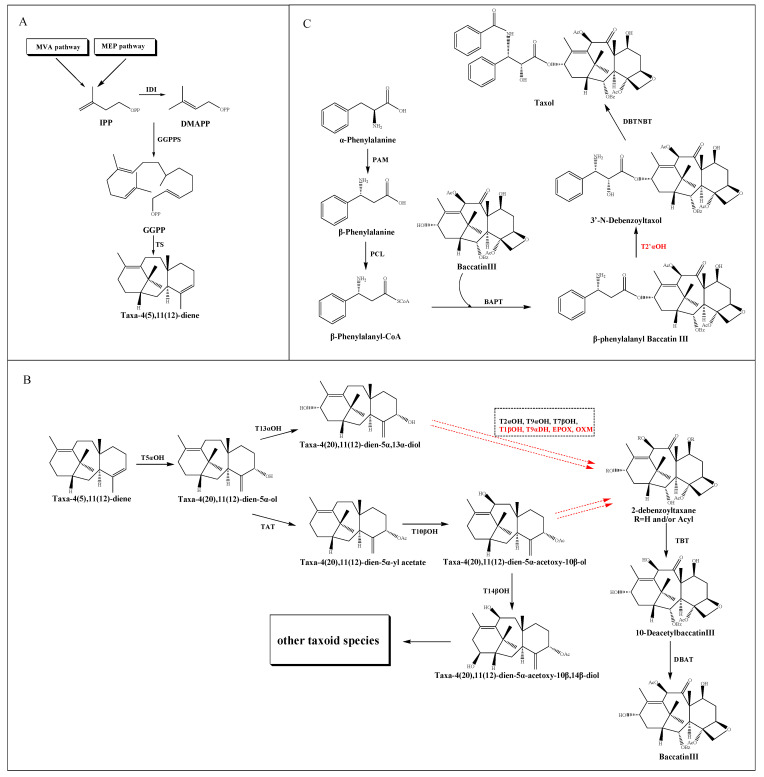
Putative taxol biosynthetic pathway. (**A**) The source and formation of the taxane core. (**B**) The modification of the core taxane skeleton. (**C**) The synthesis of the β-phenylalanyl-CoA side chain and the assembly of taxol. The solid arrows show the identified steps. The dotted arrows show the unknown steps. Steps marked by red color represent the uncharacterized enzymatic reactions. Dotted frame represents intermediate steps of the pathway where several enzymes and the order of their reactions are unknown.

The taxol biosynthetic pathway has been basically elucidated, which requires estimated 20 enzymatic steps from the common diterpenoid precursor geranylgeranyl diphosphate (GGPP) to taxol itself. As shown in Figure 1, this complex process can be divided into three parts: the source and formation of taxane core, the modification of the core taxane skeleton and the synthesis of β-phenylalanyl-CoA side chain and the assembly of taxol.

### 2.1. The Source and Formation of Taxane Core

The taxane core, namely taxadiene, derived from the cyclization of the GGPP catalyzed by taxadiene synthase (TS) [24]. In detail, GGPP is converted from three molecules of isopentenyl diphosphate (IPP) and one of dimethylallyl diphosphate (DMAPP) catalyzed by geranylgeranyl diphosphate synthase (GGPPS) [25]. IPP is considered as a common intermediate in the cytosolic mevalonic acid (MVA) pathway and the plastid 2-*C*-methyl-*D*-erythritol-4-phosphate (MEP) pathway. The conversion of IPP to DMAPP is catalyzed by a plastid IPP isomerase (IDI) [26]. Recently, the dispute over the source of IPP and DMAPP for the taxol pathway has made substantial progress. Despite evidence suggested that fosmidomycin and mevinolin could inhibit the plastid and cytosolic IPP pathways of *T. baccata* cells respectively [27,28], their selective blocking function in a *TS*-transgenic *N. benthamiana* plant suggesting that the MEP pathway supplies the bulk of the isoprenoid precursors for the taxane skeleton [18]. Moreover, the coexpression model of taxol synthesis related genes and upstream genes in *T. cuspidate* suggested a local IPP bias from the plastids [29] (Figure 1A).

### 2.2. The Modification of the Core Taxane Skeleton

After the synthesis of taxane skeleton, multiple tailoring enzymes are involved in modifying the skeleton at the C-1, C-2, C-4, C-5, C-7, C-9, C-10 and C-13 positions (Figure 1B). Firstly, a class II cytochrome P450 (CYP450) hydroxylase called taxadiene-5α- hydroxylase (T5αOH) [30] converts taxadiene into taxadien-5α-ol via hydroxylation at the C-5 position and migration of the carbon double bond. Then, the taxol pathway has been reported in two speculations: the taxadien-5α-ol is instigated by either a hydroxylation at the C-13 position to form taxadien-5α,13α-diol [31], or an acylation at C-5 position to form taxadien-5α-yl acetate [32], which could be further hydroxylated at C-10 position to form taxadien-5α-acetoxy-10β-ol [33]. Recently, a hydroxylation at the C-14 position leads to the formation of taxadien-5α-acetoxy-10β,14β-diol, but the intact structure of taxane core showed no modification at C-14 position, which implied a putative bifurcation leads to the biosynthesis of other taxoids [34]. The hydroxylation of the taxane skeleton has proven to be mediated by the CYP450 family. However, these CYP450s are selective for acetylated and polyoxygenated taxadiene substrates. Trials to produce triols from the diol intermediates using the corresponding CYP450s are currently unsuccessful.

The order of the catalytic steps after diol intermediates is not yet clear, including a series of hydroxylation at C-1, C-2, C-4, C-7 and C-9 positions, a further oxidation at C-9 position, and a formation of oxetane ring at C-4 and C-5 positions. Since the oxygenation of C-9 is believed to be an early event, while the oxygenation of C-1 happens later, Holton et al. [35] reported the order of hydroxylation should be C5, C10, C13, C9, C7, C2 and C1. However, according to the ratio of oxygenated taxoids found in *Taxus* cells, Vongpaseuth and Roberts [36] considered the order of C5, C10, C13, C2, C9, C7 and C1 may be more accurate. A series of transformations produce the hypothetical precursor 2-debenzoyltaxane, which could be further catalyzed by the enzyme taxane-2α-*O*-benzoyl transferase (TBT) to produce 10-DAB [37], one of the most important precursors for the chemical semisynthesis of taxol. Then a 10-deacetylbaccatin III-10-*O*-acetyl transferase (DBAT) could catalyze 10-DAB at the C-10 position to generate baccatin III, which is considered to be the last reaction in the formation of taxane core [38].

### 2.3. The Synthesis of β-Phenylalanyl-CoA Side Chain and the Assembly of Taxol

The β-phenylalanyl-CoA side chain is derived from α-phenylalanine, which is first converted into β-phenylalanine by phenylalanine aminomutase (PAM) [39]. Subsequently, a β-phenylalanyl-CoA ligase (PCL) [40] is predicted to convert β-phenylalanine into β-phenylalanyl-CoA. This intermediate is then attached to the C13 hydroxyl group of the taxane core by the enzyme baccatin III: 3-amino, 13-phenylpropanoyltransferase (BAPT) to produce β-phenylalanyl baccatin III [41], which is then hydroxylated at the C2’ position of the side chain, and terminal *N*-benzoylation at the C3’ position by the enzyme *N*-benzoyl transferase (DBTNBT) to produce the final product taxol [42] (Figure 1C).

### 2.4. Key Enzymes Involved in the Taxol Pathway

**Figure 2:**
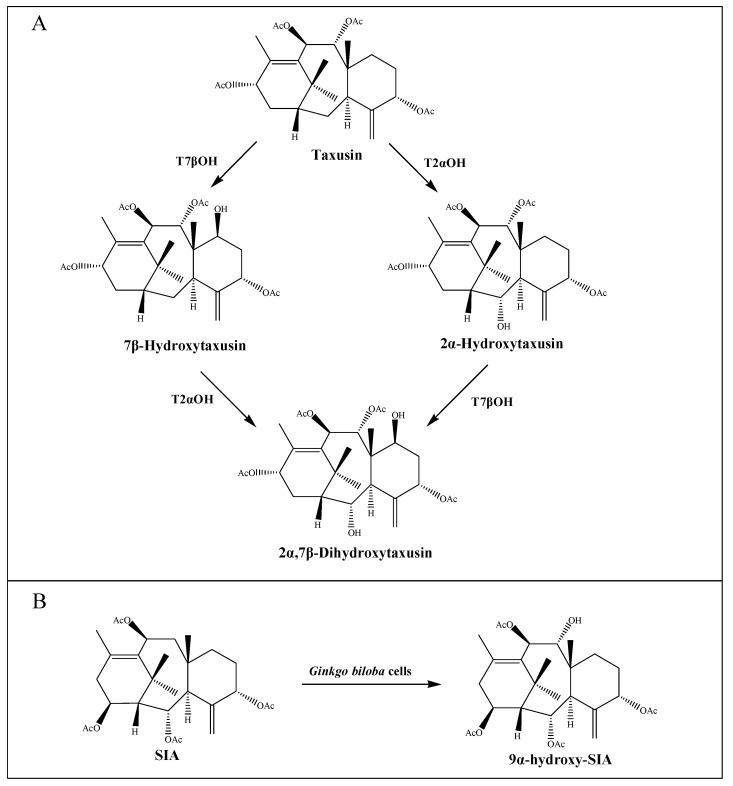
Putative intermediate steps of the taxol pathway. (**A**) T2αOH- and T7βOH-mediated hydroxylation reactions. (**B**) T9αOH-mediated hydroxylation reaction in *Ginkgo biloba* cells.

So far, sixteen enzymes involved in the taxol pathway have been well characterized (Table 1), including a novel C10 hydroxylase [30] and a putative PCL. The enzymes responsible for C-1 hydroxylation, C-2′ hydroxylation and C-9 oxidation are currently unknown, but are predicted to belong to the CYP450 family. Moreover, neither the proposed C4β, C20-epoxidase (EPOX) nor the oxomutase (OXM) responsible for the formation of oxetane ring has been identified, but potential gene candidates have been discovered through the analysis of jasmonate-induced *T. baccata* cultures [40]. Interestingly, both taxane-2α-hydroxylase (T2αOH) and taxane-7β-hydroxylase (T7βOH) could use taxusin as a substrate to form 2α-hydroxytaxusin and 7β-hydroxytaxusin, respectively. Then, these intermediates can reciprocally convert the corresponding hydroxyl products of the respective reactions to the common 2α,7β-dihydroxytaxusin [43,44] (Figure 2A). This suggested that the taxol biosynthetic pathway may not be a single linear, but a complex network of anastomosing routes that potentially have several common nodes. Moreover, the enzyme taxane-9α-hydroxylase (T9αOH) was characterized by Zhang et al. [45] in *Ginkgo biloba* cells, which can convert Sinenxan A (SIA) to form 9α-hydroxy-SIA (Figure 2B). This further implied that the process in the production of 2-debenzoyltaxane may involve transient acylation/deacylation for the purposes of trafficking and organellar targeting, or flux regulation. Such processes also greatly increase the number of biosynthetic steps and pathway complexity [46].

Recently, the differential mechanism of taxol synthesis among different tissues of *Taxus mairei* [47] and *T. cuspidate* [29], different cultivars within *T. yunnanensis* [48,49], and different *Taxus* species (*T. media*, *T. mairei* and *T. cuspidata*) [50,51] was revealed respectively by transcriptome sequencing technology, which showed huge correlations between the taxol contents and the expression levels of taxol pathway genes. Multiomics analysis further confirmed the regulation of these corresponding enzymes on taxol and its derivatives [52,53,54]. Moreover, different elicitors were used to treat *Taxus* cells. Among them, exogenous methyl jasmonate (MeJA) elicitation significantly increased the expression levels of several taxol pathway genes, such as the *Taxus* ACT genes [55,56]. The promoter sequences of related genes contained cis-elements involved in different hormones and abiotic stress responses, which may explain why these factors could stimulate taxane biosynthesis in *Taxus* species [57]. Additionally, the process was believed to be mainly mediated by a series of specific transcription factors (TF), such as MYB, bHLH, ERF, AP2 and MYC. So far, the divergence roles of two ERF TFs, TcERF12 and TcERF15 act as a repressor and activator of the *TS* gene [58], the negative regulation of TcMYC2a on genes encoding for TS, TAT, DBTNBT, T5αOH and T13αOH either directly or via ERF regulators depending on JA signaling transduction [59], and the positive regulation of TcWRKY1 on its target gene *DBAT* [60], have been gradually revealed. Moreover, the related regulatory mechanisms of a phloem-specific TmMYB3 [61], and the comprehensive analysis of the R2R3-MYB TF family [62] have also been successively reported in recent years, which promoted further understanding on taxol biosynthesis. Therefore, the increase of production of taxol could be realized by adjusting the catalytic enzymes at the key sites of the core taxane skeleton, which is also the focus and difficulty in the current synthetic biology of taxol.

## 3. Acylation Reactions Mediated by *Taxus* ACTs

The acylation reactions mediated by *Taxus* ACTs for natural taxane metabolism are common and biochemically significant, which substantially contribute to the structural and functional diversity of taxanes. These supported ACTs mainly belong to a superfamily named BAHD, which is according to the first letter of each of the first four biochemically characterized enzymes of this family, including benzylalcohol *O*-acetyl transferase (BEAT), anthocyanin *O*-hydroxycinnamoyl transferase (AHCT), anthranilate *N*-hydroxycinnamoyl/benzoyl transferase (HCBT) and deacetylvindoline 4-*O*-acetyl transferase (DAT). The BAHD family is a large group of plant-specific proteins for acylation of secondary metabolites. These proteins mainly utilize acyl-CoA as the substrate to produce small volatile esters, modified anthocyanins and constitutive defense compounds and phytoalexins [63].

### 3.1. Characterized Taxus ACTs Involved in the Taxol Pathway

At least five ACTs have been characterized to be involved in taxol pathway, including TAT, TBT, DBAT, BAPT and DBTNBT, which are grouped in the second subgroup within clade V of BAHD family [64]. These enzymes differ in substrate specificities for both acyl donors and acceptors, and they utilize acetyl-CoA, benzoyl-CoA or phenylalanoyl- CoA for *O*- and *N*-acylation of various taxanes.

#### 3.1.1. Taxadiene-5α-ol-*O*-acetyl Transferase (TAT)

The enzyme TAT catalyzes the first acylation step of taxol pathway, which converts taxadien-5α-ol to taxadien-5α-yl acetate. This reaction is a slow step for the downstream hydroxylation reactions, but not a rate-limiting step in the synthesis of core taxane skeleton. Clone TAT bears an open-reading frame (ORF) of 1317 nucleotides and encodes a deduced protein of 439 amino acids with a calculated molecular weight of 49,079. Expression of this clone in *E. coli* JM109 cells yielded the functional enzyme, which possesses the HXXXDG motif associated with catalytic activity. The deduced amino acid sequence of TAT has higher similarity (64–67%) and identity (50–56%) with other ACTs involved in different pathways of secondary metabolism in plants, but shows rather little overall homology. Moreover, the amino acid sequence information of TAT is slightly different between *T. canadensis* and *T. cuspidata* (91% identity), which may attribute to the species (subspecies) differences or to allelic variations [32].

#### 3.1.2. Taxane-2α-*O*-benzoyl Transferase (TBT)

**Figure 3:**
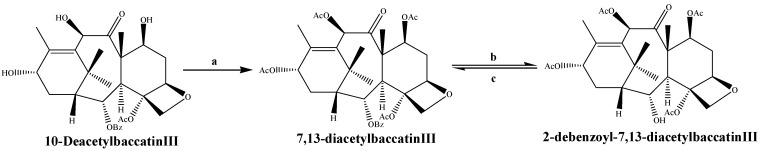
Outline of the synthesis and utilization of 2-debenzoyl-7,13-diacetylbaccatin III. Step **a** and step **b** indicate the synthetic pathway of 2-debenzoyl-7,13-diacetylbaccatin III from 10-deacetylbaccatin III. Step **c** indicates the reaction catalyzed by TBT in the presence of benzoyl-CoA.

The enzyme TBT appears to function in a late-stage acylation step of the taxol biosynthetic pathway, which catalyzes the conversion of 2-debenzoyl-7,13-diacetyl baccatin III to 7,13-diacetyl baccatin III (Figure 3). The TBT cDNA contains an ORF of 1320 nucleotides and encodes a protein of 440 amino acids with a calculated molecular weight of 50,089. Expression of this clone in *E. coli* JM109 cells afforded a functional enzyme, which possesses the HXXXDG motif associated with catalytic activity. TBT has a closer relationship with TAT and DBAT (similarity 74% and 70%, respectively; identity 68% and 64%, respectively). Moreover, the similarity and identity with other ACTs also reached 64–65% and 50–56%, respectively [37] More database retrieval with PSI-Blast from other enzymes families of *Taxus* showed that such higher similarities are universal [65].

#### 3.1.3. 10-Deacetylbaccatin III-10-*O*-acetyl Transferase (DBAT)

The enzyme DBAT, catalyzing the acetylation of the C10 hydroxyl group of 10-DAB to yield baccatin III, is considered a key rate-limiting enzyme of the taxol pathway [21]. The DBAT cDNA contains an ORF of 1320 nucleotides, which is exactly the same length as TBT, and encodes a protein of 440 amino acids with a calculated molecular weight of 49,052. A functional enzyme has been expressed in *E. coli* JM109 cells, which possesses the HXXXDG motif associated with catalytic activity. The amino acid sequence similarity and identity between DBAT and TAT are 80% and 64%, while those with other ACTs are 65–67% and 56–57%, respectively [38]. Moreover, the active sites of DBAT and residues that recognize acyl donors and taxane substrates have recently been revealed, which would be elucidated in detail in a later section.

#### 3.1.4. Baccatin III: 3-amino, 3-phenylpropanoyl Transferase (BAPT)

The enzyme BAPT catalyzes the selective 13-*O*-acylation of baccatin III with β-phenylalanoyl CoA as the acyl donor to form *N*-debenzoyl-2′-deoxytaxol (β-phenylalanyl baccatin III). The BAPT cDNA contains 1335 nucleotides and encodes a protein of 445 amino acids with calculated molecular weight of 50,546. The functional enzyme has been expressed in *E. coli* BL21(DE3) cells. The amino acid level similarity between BAPT and the four other ACTs involved in taxol pathway ranged from 71% to 74%. However, The BAPT sequence is the only one of the five that contains a G_163_XXXDA_168_ motif instead of the typical HXXXDG, or less frequent HXXXDA motif. The Gly-163 for His-163 substitution in BAPT would likely disrupt a suggested catalytic function involved in acyl group transfer. Interestingly, the free β-amine of the CoA ester cosubstrate in this instance could, through hydrogen bonding, function as a surrogate intramolecular general acid/base in place of the normal histidine at this position [41].

#### 3.1.5. *N*-benzoyl Transferase (DBTNBT)

**Figure 4:**
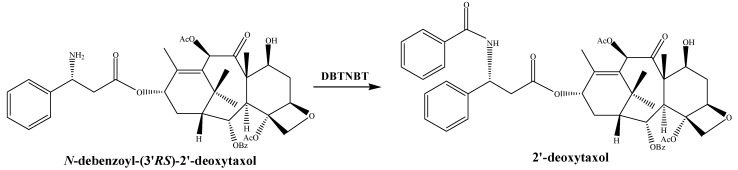
The enzyme DBTNBT catalyzes the surrogate substrate *N*-debenzoyl-(3′*RS*)-2′-deoxytaxol with benzoyl-CoA to form 2′-deoxytaxol.

The enzyme DBTNBT catalyzes the stereoselective coupling of the surrogate substrate *N*-debenzoyl-(3′*RS*)-2′-deoxytaxol with benzoyl-CoA to form predominantly one 3′-epimer of 2′-deoxytaxol (Figure 4). The properties of this enzyme indicated that it could transfer a benzoyl group to the amino group of side chain of *N*-debenzoyl-taxol, which is considered the last step of taxol pathway, because the DBTNBT has a substrate preference for *N*-debenzoyl-taxol rather than *N*-debenzoyl-2′-deoxy-taxol based upon tests with both substrates [66]. The DBTNBT cDNA contains an ORF of 1323 nucleotides encoding a protein of 440 amino acids with a calculated molecular weight of 49,040. Expression of this clone in *E. coli* BL21(DE3) cells yielded the functional enzyme, which possesses the conserved HXXXDG motif. The deduced amino acid sequence of DBTNBT has a closer relationship with TBT (60% identity, 69% similarity) [42].

### 3.2. Other Taxus ACT Genes Involved in the Taxane Metabolism

It is possible that the acylation steps may help to contributing to the formation of functional and structural diversity of taxanes and the regulation of taxol pathway flux, which is supported by the many acyltransferase genes responsible for numerous structural side chain modifications found among taxoid variants. Croteau et al. [23] reported that except for the five defined ACTs of the taxol pathway, 10 other distinct ACTs were predicted to be responsible for the production of numerous taxoid side chain structural and regiochemical variants. Chau et al. [67] cloned a new TAT gene (TAX19) that was capable of acetylating taxadien-5α-ol with activity comparable to that of the original, but exhibited different regiospecificities, preferentially acetylating the positions at C5 and C13. Two other taxoid-*O*-acetyl transferase genes (TAX9 and TAX14) were isolated by Hampel et al. [68], which appear to act exclusively on partially acetylated taxoid polyols to divert the taxol pathway to side-route metabolites. The native *N*-debenzoyl-2′-deoxypaclitaxel: *N*-benzoyltransferase (NDTBT), isolated from *Taxus* plants, transfers a benzoyl group from the corresponding CoA thioester to the amino group of the β-phenylalanine side chain of *N*-debenzoyl-2′-deoxypaclitaxel, which is purportedly on the taxol pathway [69]. Moreover, Kuang et al. [29] sequenced *Taxus cuspidata* transcriptomes with next generation sequencing (NGS) and third generation sequencing (TGS) platforms and identified seven BAHD ACT genes as potential lead candidates for the formation of the significant precursor 2-debenzoyltaxane based on phylogenetic and coexpression analysis. However, this requires more investigation because the acylation of the poly-hydroxylated substrate is likely to occur during the formation of a heptaol intermediate. These combined results indicated that the acylation may control the synthesis and yield of taxol, theoretically by limiting the accumulation of intermediates for taxol biosynthesis, and providing precursors for other taxoid species likely also possessing distinct biological roles. Therefore, the mining and analysis of specific *Taxus* ACTs for taxane acylation are the key to further improve the understanding of network of taxane metabolism.

### 3.3. Formation of Taxane Core Mediated by Taxus ACTs

Kusano et al. [70] reported that the specialized *Taxus* BAHD proteins evolved from a common lineage, and form a *Taxus*-specific clade, containing all five characterized ACTs involved in the taxol pathway, and other *Taxus* proteins of unknown function. Similar results have also been reported by Kuang et al. [29]. It has been strongly considered that the neofunctionalization is induced by the acquisition of promiscuous enzymatic activity allowed by the increase in the number of gene copies during plant evolution, which resulted in the synthesis of new metabolites and establishment of biosynthetic pathways in the plant lineage [71]. The clustering of all specialized *Taxus* ACTs suggested an important role of the considerable expansion of *Taxus* BAHD family members for the taxane metabolism and development of multiple specialized traits within the *Taxus* species. Walker et al. [32] reported that the enzyme TAT had the DBAT activity, which was 13.2% that of DBAT. While DBAT isolated from different *Taxus* species exhibited acetylation activity against 10-DAB and 10-deacetyltaxol, a de-glycosylated derivative of 7-b-xylosyl-10-deacetyltaxol [72]. Moreover, NDTBT could transfer hexanoyl, acetyl and butyryl more rapidly than benzoyl from the CoA donor to taxanes with isoserinoyl side chains, whereas *N*-debenzoyl-2’-deoxytaxol was more rapidly converted to its *N*-benzoyl derivative than to its *N*-alkanoyl or *N*-butenoyl congeners [69]. These combined results may represent the evolutionary footprint of *Taxus* BAHD proteins that acquires new functionalities through the alterations of substrate and product specificities, and resulting in the production of unique taxane compounds. Since the rates of evolution for genes involved in plant specialized metabolism are greater than that in primary metabolism, it also provided valuable information to explain the formation of taxane core from the exon–intron sequence of the *Taxus* BAHD family members [70].

### 3.4. Acylation Mechanism Mediated by Taxus ACTs

Recently, the crystal structure of vinorine synthase, a member of the BAHD family, was obtained by Ma et al. [73], who made a major breakthrough in the understanding of the structural and functional characteristics of ACTs within the plant kingdom. Li et al. [72] generated a three-dimensional structure of DBAT and identified its active site using alanine scanning, and designed a double DBAT mutant (DBAT G38R/F301V) with a catalytic efficiency approximately six times higher than that of the DBAT wildtype (WT), which improved an in vitro one-pot conversion of 7-b-xylosyl-10-deacetyltaxol to taxol. Moreover, the activity essential residues of the enzyme DBAT, and the acylation mechanism from its natural substrate 10-DAB and acetyl CoA to baccatin III were investigated by You et al. [74]. Among them, residues H162, D166 and R363, located in the catalytic pocket of the enzyme, were important for DBAT activity; and residues S31 and D34 from motif SXXD, D372 and G376 from motif DFGWG were important for acylation. Based on the above results, You et al. [75] redesigned the active sites of enzyme DBAT (H162A/R363H, D166H, R363H and D166H/R363H), which displayed 3, 15, 26 and 60 times higher catalytic activities than that of the WT, respectively, and these mutants could transfer acetyl group from unnatural acetyl donor (e.g., vinyl acetate, sec-butyl acetate, isobutyl acetate, amyl acetate and isoamyl acetate) to 10-DAB. These studies also provide a reference for the comprehensive elucidation of taxane acylation mechanism and the synthesis and regulation of taxol in vitro and in vivo.

## 4. Conclusions and Perspectives

Taxol is one of the most effective anticancer drugs, which could be used to treat a variety of cancers. However, the industrial production of taxol still relies on chemical synthesis after the precursor is extracted from the *Taxus* trees, which seriously affected the survival and reproduction of *Taxus* species. Therefore, it is essential to explore alternative methods and increase the taxol yield, which mainly depends on the understanding of the taxol biosynthetic pathway and related enzymes in detail. So far, the basic framework of taxol pathway has been elucidated, including the source and formation of the taxane core and the process of the downstream synthetic pathway, and most of the enzymes involved have been characterized. Only the order of hydroxylation reactions during the modification of the core taxane skeleton and the substrate specificity of related enzymes remain unclear. Moreover, this process may involve transient acylation/deacylation, which greatly increase the pathway complexity. Recently, the characteristics of *Taxus* ACTs in guiding the evolution of taxol pathway, promoting the diversity of taxane compounds and regulating the synthesis and yield of taxol are continuously reported. Several ACTs involved in taxane metabolism have also been identified, which contributed to the understanding of taxane formation. However, the knowledge on the establishment of the entire taxane metabolic regulatory network associated with the acylation reactions is still in its infancy. For *Taxus* ACTs, the typical conserved characteristics, differences in substrate specificity and the different enzymatic forms and activities, reflect the versatility of the specialized *Taxus* BAHD family for taxol synthesis. Moreover, the utilization of heterologous expression systems and the use of alternative substrates have greatly enriched the research on the mining and analysis of *Taxus* BAHD family members. With the popularization of the third-generation transcriptome sequencing technology and the growing maturity of biochemical and molecular genetics studies, the understanding of *Taxus* BAHD family contributing to specialized development of taxane metabolism will be further improved. In our opinion, except for the identification of unknown enzymes on the taxol pathway, the subsequent work should focus on the completion of taxane acylation mechanism mediated by *Taxus* ACTs, especially the acylation process involved in the rate-limiting steps of taxol pathway and the specific recognition modes of the taxane substrates. This will help to further understand the taxol biosynthetic pathway and mass production of taxol in vivo and in vitro, thereby alleviating the contradiction of taxol between the supply and demand.

## Figures and Tables

**Table 1:** Characterized key enzymes in the taxol pathway.

Enzyme Abbreviation	Size (kDa)	Probable Localization	GenBank Number
Geranylgeranyl diphosphate synthase GGPPS	42	Plastids	AF081514
Taxadiene synthase TS	98	Plastids	AY364469
Taxadiene-5α-hydroxylase T5αOH	56	Endoplasmic reticulum	AY289209
Taxane-10β-hydroxylase T10βOH	56	Endoplasmic reticulum	AF318211
Taxane-10β-hydroxylase ^a^ T10βOH	55	Endoplasmic reticulum	AY563635
Taxadiene-13α-hydroxylase T13αOH	54	Endoplasmic reticulum	AY056019
Taxane-2α-hydroxylase T2αOH	55	Endoplasmic reticulum	AY518383
Taxane-9α-hydroxylase T9αOH	55	Endoplasmic reticulum	KF773141
Taxane-7β-hydroxylase T7βOH	56	Endoplasmic reticulum	AY307951
Taxadiene-5α-ol-*O*-acetyl transferase TAT	49	Cytosol	AF190130
Taxane-2α-*O*-benzoyl transferase TBT	50	Cytosol	AF297618
10-deacetylbaccatin III-10-*O* acetyl transferase DBAT	49	Cytosol	AF193765
Baccatin III: 3-amino, 13-phenylpropanoyltransferase BAPT	50	Cytosol	AY082804
*N*-benzoyl transferase DBTNBT	49	Cytosol	AF466397
Phenylalanine aminomutase PAM	76	Cytosol	AY582743
β-phenylalanyl-CoA ligase ^b^ PCL	59	Cytosol	KM593667

^a^ A second taxane-10β-hydroxylase has also been found; ^b^ The PCL listed here is a putative candidate isolated from *T. baccata* cultures.
